# Xanthan gum-based edible coating effectively preserve postharvest quality of ‘Gola’ guava fruits by regulating physiological and biochemical processes

**DOI:** 10.1186/s12870-024-05117-1

**Published:** 2024-05-23

**Authors:** Shaista Gull, Shaghef Ejaz, Sajid Ali, Muhammad Moaaz Ali, Hasan Sardar, Muhammad Azam, Honghong Deng, Ahmed Fathy Yousef, Abdulwahed Fahad Alrefaei, Mikhlid H. Almutairi

**Affiliations:** 1 Department of Horticulture, Bahauddin Zakariya University, Multan, Punjab 60800 Pakistan; 2 College of Horticulture, Fujian Agriculture and Forestry University, Fuzhou, 350002 China; 3 Pomology Laboratory, Institute of Horticultural Sciences, University of Agriculture, Faisalabad, 38040 Pakistan; 4 Department of Horticulture, College of Agriculture, University of Al-Azhar (Branch Assiut), Assiut, 71524 Egypt; 5 Department of Zoology, College of Science, King Saud University, P.O. Box 2455, Riyadh, 11451 Saudi Arabia

**Keywords:** Guava postharvest, Polysaccharide coating, Cell wall degradation, Pectin, Hemicellulose, Antioxidant activities, Hydrocolloid

## Abstract

**Background:**

Guava is a fruit prone to rapid spoilage following harvest, attributed to continuous and swift physicochemical transformations, leading to substantial postharvest losses. This study explored the efficacy of xanthan gum (XG) coatings applied at various concentrations (0.25, 0.5, and 0.75%) on guava fruits (Gola cultivar) over a 15-day storage period.

**Results:**

The results indicated that XG coatings, particularly at 0.75%, substantially mitigated moisture loss and decay, presenting an optimal concentration. The coated fruits exhibited a modified total soluble soluble solids, an increased total titratable acidity, and an enhanced sugar-acid ratio, collectively enhancing overall quality. Furthermore, the XG coatings demonstrated the remarkable ability to preserve bioactive compounds, such as total phenolics, flavonoids, and antioxidants, while minimizing the levels of oxidative stress markers, such as electrolyte leakage, malondialdehyde, and H_2_O_2_. The coatings also influenced cell wall components, maintaining levels of hemicellulose, cellulose, and protopectin while reducing water-soluble pectin. Quantitative analysis of ROS-scavenging enzymes, including superoxide dismutase, peroxidase, catalase, and ascorbate peroxidase, revealed significant increases in their activities in the XG-coated fruits compared to those in the control fruits. Specifically, on day 15, the 0.75% XG coating demonstrated the highest SOD and CAT activities while minimizing the reduction in APX activity. Moreover, XG coatings mitigated the activities of fruit-softening enzymes, including pectin methylesterase, polygalacturonase, and cellulase.

**Conclusions:**

This study concludes that XG coatings play a crucial role in preserving postharvest quality of guava fruits by regulating various physiological and biochemical processes. These findings offer valuable insights into the potential application of XG as a natural coating to extend the shelf life and maintain the quality of guava fruits during storage.

## Background

Guava (*Psidium guajava* L.) hails from tropical regions and is esteemed for its delightful flavor and nutritional benefits. When comparing nutritional content, the red guava surpasses the tomato, boasting notably higher levels of fiber, lycopene, and vitamin C [1, 2]. Brazil is the foremost global consumer of guava, annually producing 424,000 tons of this fruit. The states of Pernambuco and São Paulo play a pivotal role, contributing to a substantial 68% of the national production [3]. Characterized as a climacteric fruit, guava exhibits rapid physiological metabolism, a high respiratory rate, and swift maturation, necessitating a brief commercialization period [4].

Reactive oxygen species (ROS) generated within stored guavas are accountable for inducing oxidative stress-related damage to cell wall polysaccharides (CWPs), culminating in their breakdown [5]. Key components of the cell wall, such as cellulose, hemicellulose, and pectin-based substances, undergo depolymerization when cell wall hydrolase enzymes are activated, leading to softening of the fruit [6]. Various hydrolytic enzymes, including pectin methylesterase (PME), polygalacturonase (PG), and cellulase (CS), have been identified as contributors to the breakdown of cell wall structures and subsequent softening of fruit tissues [7]. In particular, PME and PG play crucial roles in degrading pectin-based substances. Plant molecular elicitors induce the de-esterification of pectin from galacturonic acid, while PG facilitates the hydrolysis of polygalacturonic acid. This process results in the loosening of the cell wall and a reduction in fruit firmness [8]. Consequently, it is imperative to adopt environmentally friendly and effective treatments that can hinder the actions of hydrolase enzymes, thereby postponing the softening of harvested guavas and preserving their overall eating quality.

Employing low storage temperatures is a widely utilized strategy to extend the shelf life of fruits [9]. However, while effective, refrigeration can be expensive and is limited for certain tropical fruits, such as guava fruits, due to their susceptibility to chilling injury [10]. Recently, there has been a surge in the development of new postharvest technologies designed to increase the shelf life of fruits. These innovations are in response to consumer demands, which prioritize factors such as food safety, the absence of chemical preservatives, reasonable pricing, and environmentally friendly practices. One such method involves the application of edible coatings [11]. These thin, imperceptible membranes are directly applied to the fruit surface. They can transport natural additives that play a vital role in preserving the freshness of food. These additives bolster the protective properties of the fruit’s outer layer, preventing issues such as water loss, physical damage, and microbial decay. Additionally, they contribute to the fruit’s glossy appearance [12–14]. This technology presents a promising opportunity for cost-effective and efficient implementation in food processing facilities.

Xanthan gum (XG), derived from *Xanthomonas campestris*, is primarily employed for its stabilizing, thickening, and emulsifying properties. Its remarkable resistance to enzyme degradation makes it effective as a coating for both fresh-cut and whole fruits, providing a lasting impact [15]. Consequently, incorporating XG into food products can achieve the dual objectives of extending shelf life and imparting a glossy appearance simultaneously [16]. Previous research has explored the application of XG coatings on various fresh-cut fruits, including pears, strawberries, tomatoes, bananas, jujubes, and grapefruit slices, during storage [15, 17–21]. However, there is a notable gap in specific information regarding the use of XG on freshly harvested guava fruit. Therefore, our objective was to investigate the impact of XG coating on factors such as fruit moisture loss, oxidative stress, softening, and biochemical quality in fresh guavas.

## Materials and methods

### Guava fruits

Unripe and physiologically mature pale green guava fruits of the ‘Gola’ cultivar were freshly harvested in the morning from an orchard situated approximately 4 km away from Bahauddin Zakariya University (BZU), Multan. Rigorous sorting was conducted to ensure uniformity in color, size, and maturity stage, and only fruits free from any visible damage or physiological decay were selected. Subsequently, the fruits were promptly transported to the Postgraduate Laboratory within the Department of Horticulture at BZU, Multan. Upon arrival, the fruits underwent a meticulous cleaning process, which included washing, disinfection (using 0.01% NaOCl for 2 min), and thorough rinsing. Following these procedures, the fruits were subjected to air drying at 25 ± 2 °C for 90 min.

### XG preparation and application

The coating solution was prepared by mixing 2.5, 5.0–7.5 g of xanthan gum (CAS No. 11,138–66–2; Sigma Aldrich, St. Louis, USA) powder in 950 mL of autoclaved distilled water at 50 °C under magnetic stirring for 3 h. Afterwards, 1% glycerol as the plasticizer and 0.25% Tween-20 as the surfactant were added before making the final volume of the solution to 1 L with autoclaved distilled water [6]. To eliminate potential air bubbles, the resulting coating solution was sonicated for approximately 30 min. The solution was subsequently allowed to stand at room temperature for an additional 60 min before being applied to the guava fruits.

The guava fruits were coated with XG at various concentrations, specifically 0.25, 0.5, and 0.75%, while noncoated fruits were used as the control. Each replication of a treatment contained 12 fruits. The fruits were immersed in the respective coating solutions for 3 to 5 min and subsequently air-dried under ambient conditions (∼ 20 °C, > 75%). Following the coating process, the fruits were stored at 20 ± 1 °C and 85–90% relative humidity. 6 to 8 fruits from each replication of a treatment were sampled for analysis on each sampling day i.e., on days 3, 6, 9, 12, and 15 after coating. The experiment was carried out according to a completely randomized design with four replications.

On each sampling day, after measuring fruit moisture loss, decay incidence, respiration rate, ethylene rate, total soluble solids, total titratable acids, ascorbic acid, and electrolyte leakage, the homogenized mixture of fruits’ pulp was frozen using liquid nitrogen and stored at -80℃ for further use.

### Fruit moisture loss and decay incidence

The moisture loss and decay incidence in the fruits were assessed following the procedures outlined by El-Gioushy et al. [12], with minor adjustments. To determine moisture loss, the percentage difference between the initial day and sampling day was calculated. Decay incidence was determined by visually inspecting the presence of decayed or rotten fruits and expressing it as a percentage.

### Total soluble solids, total titratable acids, sugar-acid ratio and ascorbic acid

Total soluble solids were measured in fruit juice using a digital refractometer (PAL-1, Atago, Japan), while titratable acidity was measured by titrating juice with NaOH [22]. Briefly, 10 g of fruit pulp was grinded with 90 mL of water for extraction and filtered through two-layered muslin cloth. The filtrate was thus titrated with 0.1 N NaOH to pH 8.1 and expressed as percentage of citric acid. The sugar-acid ratio was calculated by dividing the total soluble solids by the titratable acids [23]. An earlier method was used to measure ascorbic acid content [24]. Briefly, oxalic acid at a concentration of 0.4% was added to 10 mL of fruit juice. After that, an aliquot of 5 mL was titrated with 2,6 dichloroindophenol. L-ascorbic acid was used as the standard, and the concentration of ascorbic acid was expressed in milligrams per 100 g of fresh weight (mg·100 g^−1^ FW).

### Total phenolics, flavonoids and antioxidants

The phenolic content was quantified using the method of Singleton and Rossi [25], and the findings are expressed as mg·kg^−1^. Total flavonoids were assessed according to the detailed protocol of Zhi et al. [26] and are expressed as g·kg^−1^. Total antioxidant concentrations were measured according to the methods of Krings and Berger [27], and the results are expressed as mmol Trolox·100 g^−1^.

### Electrolyte leakage, malondialdehyde and hydrogen peroxide contents

The electrolyte/ion leakage of guava fruits was assayed following the methods of Yang et al. [28]. Fruit tissue Sect. 3 mm in thickness and 10 mm in diameter were cut and put into deionized water for 30 min at 25 °C. The conductivity was measured with an EC meter (HI-98,304, Hanna Instruments, Inc., Mauritius) after the fruit discs were boiled in water at 100 °C. Relative ion leakage (electrolyte leakage) was expressed as a percentage [23].

To determine the amount of fruit malondialdehyde (MDA) content, a sample of 1 g was homogenized in 15 mL of 10% trichloroacetic acid and then centrifuged at 10,000 × *g* for 20 min. After the reaction of the supernatant with 2 mL of 0.6% 2-thiobarbituric acid, the absorbance of the supernatant was measured at 450, 532 and 600 nm [29], and the MDA concentration was expressed as mM·kg^−1^ FW.

The method previously developed by Velikova and Loreto [30] was used to calculate the H_2_O_2_ concentration. After homogenizing the 1 g sample in trichloroacetic acid at a concentration of 0.1% (1 mL), it was centrifuged at 12,000 × *g* for 15 min. 0.5 mL of the supernatant was combined with 0.5 mL of phosphate buffer (with a pH of 7.0 and a concentration of 50 mmol/L) and 1 mL of potassium iodide at a concentration of 1 M. Finally, at a wavelength of 390 nm, the absorbance was measured through a spectrophotometer (UV-1602, BMS, QC, Canada), and the findings are presented in µM·kg^−1^ FW.

### Fruit respiration and ethylene production rates

An F-950 digital gas analyzer was used (Felix Inst., USA) to determine the respiration rate and ethylene production rate in guavas [6]. Briefly, 2–3 fruits of known weight were put in a sealed glass jar (with known volume) for 60 min at 25 °C. The readings of respiration rate and ethylene production rate were measured from the headspace (through the insertion of a probe). The concentrations of respiration and ethylene were expressed as ml·CO_2_·kg^− 1^·h^− 1^ and µl·kg^− 1^·h^− 1^, respectively.

### Hemicellulose, cellulose, water soluble pectin and protopectin

The contents of hemicellulose, cellulose, water soluble pectin (WSP) and protopectin in stored guava fruits were determined using the previously described methods of Wang et al. [31]. To extract cell wall material, 30 g of frozen fruit pulp was meticulously ground with 200 mL of 80% ethanol (*v/v*). The mixture was heated for one hour and subsequently centrifuged at 2000× *g* for 10 min. The residue was washed three times with 80% ethanol, followed by a 10-hour soaking period in 90% C_2_H_6_OS (*v/v*). After repeating the filtration process, the remaining substance was washed three times with 100 mL of acetone. Vacuum drying at 40 °C was subsequently performed, and the resultant material was identified as the cell wall material. Next, 300 mg of the cell wall material was homogenized in a 10 mL solution of acetic acid buffer (50 mM, pH 6.5) and subjected to centrifugation at 10,000× *g* at 4 °C for 10 min. The sediment, obtained after removing the liquid, was dissolved in 10 mL of acetate buffer (50 mM, pH 6.5) containing 2 mM cyclohexane-trans-l,2-diamine tetraacetate. This mixture was stirred continuously for 6 h and then centrifuged at 10,000× *g* at 4 °C for 10 min. Following the dissolution of the lower precipitate in Na_2_CO_3_ (50 mM) with 2 mM cyclohexane-trans-l, 2-diamine tetraacetate, the mixture was stirred for 6 h and centrifuged at 10,000× *g* at 4 °C for 10 min. The resulting residue was dissolved in a 10 mL solution of 4 mM NaOH, which included 100 mM NaBH_4_, and then centrifuged for 10 min at 10,000× g. The sediment, identified as cellulose materials, was utilized to determine the cellulose and hemicellulose content, with measurements expressed in mg·kg^− 1^ fresh weight (FW).

A 1 g composite fruit sample was boiled in 25 mL of 95% ethanol (*v/v*) for 30 min. After cooling, the homogenate was subjected to centrifugation at 8000× *g* and 4 °C for 15 min. The resulting sediment was dissolved in 25 mL of 95% ethanol (v/v) and subjected to boiling and centrifugation using the previously mentioned procedure; this process was repeated three times. Subsequently, the residue was thoroughly mixed with 20 mL of deionized water and gently warmed at 50 °C in a water bath for 30 min. The homogenate was then centrifuged at 8000× *g* for 15 min, and the collected liquid was used for measuring the WSP. The remaining sediment was dissolved in 25 mL of 0.5 M H_2_SO_4_ and boiled for 1 h. The resulting homogenate was centrifuged at 8000× *g* for 15 min, and the supernatant was utilized to estimate the protopectin content. The protopectin and WSP contents were expressed as g·kg^− 1^ fresh weight (FW).

### Assays of the activity of ROS-scavenging enzymes

Enzyme proteins from the fruit tissues (1 g from a composite sample) were extracted in 2 mL of phosphate buffer (50 mmol·L^−1^) at pH 7.2 and homogenized with a mortar and pestle. The homogenized samples were centrifuged at 12,000 × *g* for 5 min. The supernatant was used as the enzyme extract for the following enzymes.

#### Superoxide dismutase

In a test tube, phosphate buffer (with a pH of 5 and a concentration of 50 mmol·L^−1^), distilled water, methionine and 100 µL of nitro-blue tetrazolium (NBT) were combined with the enzyme extract. Afterwards, 100 µL of riboflavin was added to the reaction mixture described above and the test tubes were exposed to 15 W fluorescent light for the next 15 min. On a spectrophotometer, absorbance readings were obtained at 560 nm at intervals of 0, 30, 60 and 90 s, and the means of these readings were utilized in the final computation [32]. One unit of the activity of SOD enzyme was defined as “the quantity of enzyme that led to 50% inhibition of NBT photoreduction”.

#### Peroxidase

In a test tube, phosphate buffer (with a pH of 5), the enzyme extract, H_2_O_2_ (40 mmol·L^−1^) and guaiacol (20 mmol·L^−1^) were mixed together. On a spectrophotometer, absorbance readings were obtained at 470 nm at intervals of 0, 30, 60 and 90 s, and the mean of these readings was utilized in the final computation [33]. One unit of the activity of POD enzyme was defined as “the change in the absorbance in 0.01 units min^− 1^”.

#### Catalase

A sample of the enzyme extract, 20 mmol·L^−1^ hydrogen peroxide, and phosphate buffer at a pH of 5 were mixed in a test tube. On a spectrophotometer set to 240 nm, the absorbance of the reaction mixture was measured at intervals of 0, 30, 60, 90 and 120 s; the mean of these measurements was utilized in the final calculations [33]. One unit of the activity of CAT enzyme was defined as “the change in the absorbance in 0.01 units min^− 1^”.

#### Ascorbate peroxidase

In a test tube, 0.5 mmol·L^−1^ L-ascorbate, 0.1 mmol·L^−1^ hydrogen peroxide, 50 mmol·L^−1^ phosphate buffer, pH 5, and 100 µL of the enzyme extract were mixed together. After the reaction mixture was transferred to a cuvette, the absorbance was measured at 290 nm at intervals of 0, 30, 60, and 90 s [34]. One unit of the activity of APX enzyme activity (U mg^− 1^ protein) was defined as “the quantity of enzyme that led to the oxidation of 1 µmol of ascorbate min^− 1^”.

### Activity assay of fruit-softening enzymes

Extraction was performed according to the methods of Saleem et al. [35]. One gram of homogenous guava sample was ground in cold sodium acetate (pH 5.5) followed by standing at 4 °C for 15 min and centrifugation at 12,000 × *g* for 30 min. The supernatant was used for the following analyses.

#### Pectin methylesterase

The activity of pectin methylesterase (PME) was determined by using, with minor modifications, the procedure of Hagerman and Austin [36]. Two milliliters of pectin (0.5%), 0.01% bromothymol blue, 0.75 mL of deionized water and 0.1 mL of crude enzyme extract were used in the pectin methylesterase assay. The absorbance at 620 nm was used to determine the enzyme activity as U·mg^−1^ (or µM·min^−1^·mg^−1^) protein. The units of PME activity were expressed as µ-moles of H^+^ released per min mg protein at given conditions.

#### Polygalacturonase

The method described by Ge et al. [37] was used to measure the activity of polygalacturonase (PG). The assay mixture included 0.5 mL of sodium acetate buffer at a pH of 4.5, 0.4 mL of pectin at a concentration of 1% and 0.1 mL of crude enzyme extract. After the reaction with dinitro-salicylic acid, the mixture was heated at 100 °C for 5 min before being left to incubate for further an hour at 37 °C. After absorbance was measured at 540 nm, the enzyme activity was subsequently expressed in units of U·mg^−1^ protein. PG activity was expressed as µmoles of galacturonsyl reducing groups liberated per minute per mg protein.

#### Cellulase

The cellulase activity was measured in accordance with the methodology of Deng et al. [38] with minor modifications. After incubating the supernatant at 37 °C for one hour, 0.1 mL of the extract was added to a cellulase activity test mixture that consisted of 0.4 mL of 1% (w/v) carboxy methylcellulose and 0.5 mL of 0.1 M sodium acetate buffer with a pH of 5.0. The mixture was heated for 5 min at 100 °C and then cooled to room temperature. The enzyme activity was reported as U·mg^−1^ protein after the absorbance was read at 540 nm on UV-VIS spectrophotometer (Carry 60, Agilent, USA). One unit of cellulase activity was defined as the amount of the enzyme that catalyzed the formation of one µ-mole reducing groups per hour per mg protein.

### Sensory/organoleptic evaluation

Organoleptic evaluation of the aroma, taste and overall acceptability of the fruits was performed by panelists (aged 25–35 years) using a sensory scale developed earlier [39–41]. The fruit samples were randomly arranged and blindly labeled for presentation to the panel. Aroma, taste and overall acceptability were evaluated with a 1 to 9 hedonic scale (1 = dislike extremely; 5 = neither like nor dislike; 9 = like extremely).

### Data analysis

The data collected at each sampling interval were analyzed with one-way analysis of variance using the statistical software “Statistix 8.1”. Treatment means were differentiated with Fisher’s least significant difference (LSD) test (*p* ≤ 0.05). The studied variables were further subjected to Pearson (*n*) correlation analysis using http://www.bioinformatics.com.cn, an online platform for data analysis and visualization.

## Results

### Fruit moisture loss and decay incidence

**Fig. 1:**
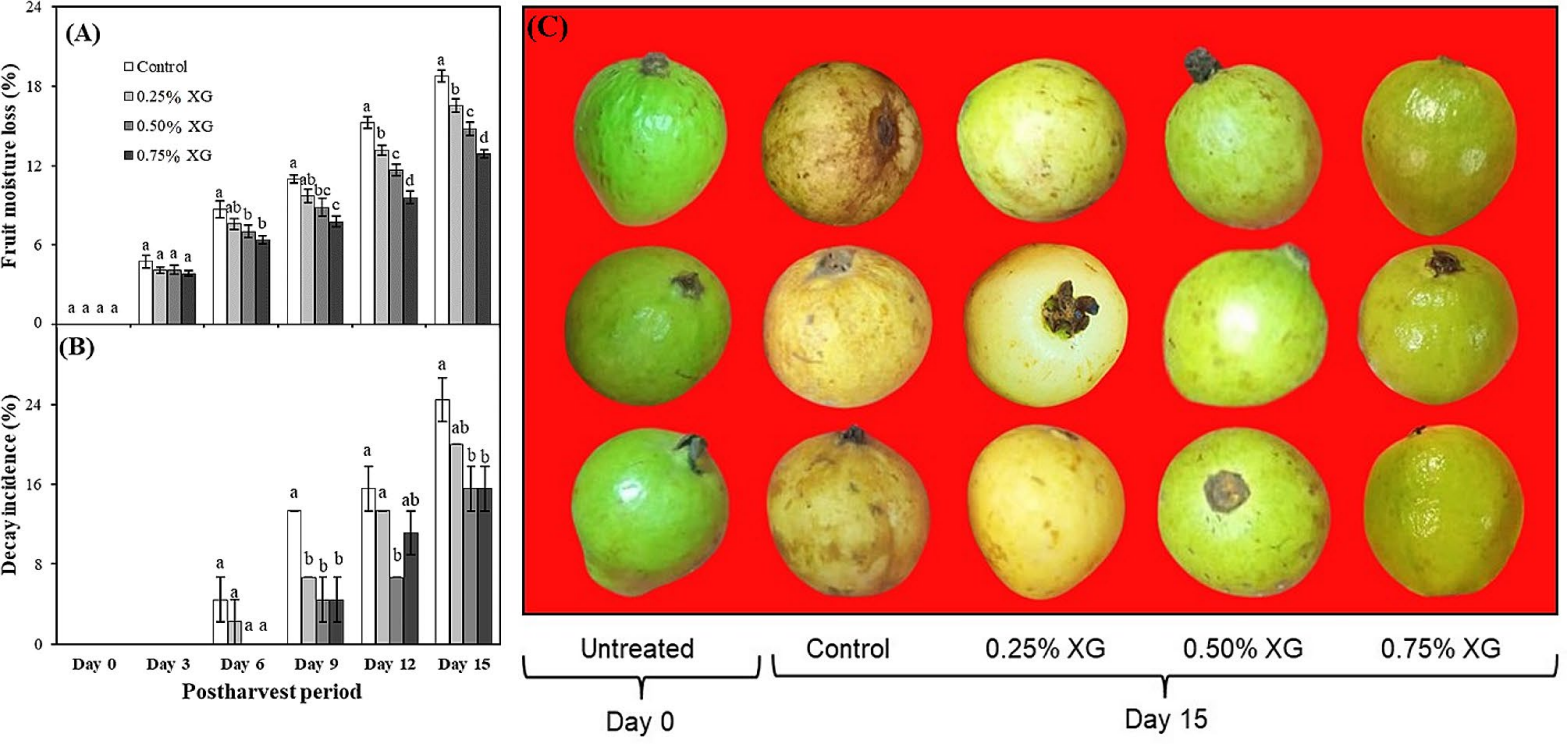
The impact of xanthan gum (XG)-based coatings on moisture loss (**A**), decay incidence (**B**) and visual appearance of harvested guava fruits. Each bar corresponds to the mean ± SE (*n* = 4). Statistical analysis using Fisher’s LSD revealed significant differences among coating treatments on each sampling day, denoted by different small letters (*p* ≤ 0.05)

The duration of storage and the application of XG coatings exerted a notable influence on the moisture loss (%) in harvested guava fruits (Fig. 1). As the storage period progressed from day 0, the moisture loss exhibited an upward trend in both the coated and uncoated guava fruits, with a particularly significant increase observed in the uncoated fruits. By the 15th day of storage, guava fruits coated with 0.75% XG demonstrated a remarkable 31.35% reduction in moisture loss compared to uncoated fruits (Fig. 1A). Concurrently, the incidence of decay (%) also escalated with prolonged storage. Notably, XG coatings played a significant role (*p* ≤ 0.05) in reducing decay incidence in guava fruits, irrespective of the applied concentration. On the 15th day of storage, XG-coated fruits exhibited a substantial 18–36% decrease in decay incidence compared to uncoated guava fruits, with the lowest incidence observed in fruits coated with 0.50–0.75% XG (Fig. 1B, C).

### Total soluble solids, total titratable acidity, sugar-acid ratio and ascorbic acid content

**Fig. 2:**
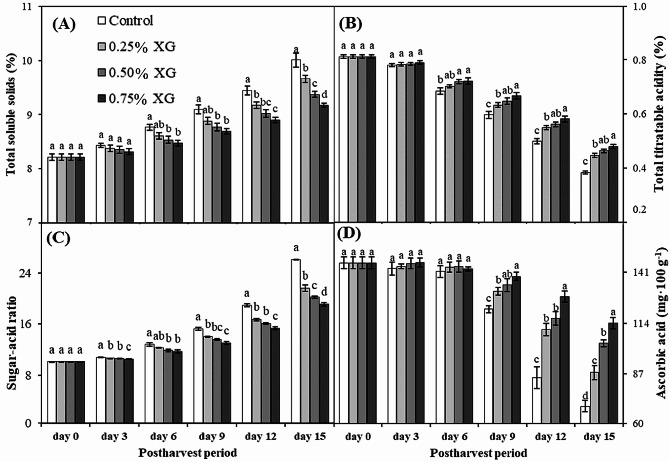
The impact of xanthan gum (XG)-based coatings on total soluble solids (**A**), total titratable acidity (**B**), sugar-acid ratio (**C**) and ascorbic acid content (**D**) in harvested guava fruits. Each bar corresponds to the mean ± SE (*n* = 4). Statistical analysis using Fisher’s LSD revealed significant differences among coating treatments on each sampling day, denoted by different small letters (*p* ≤ 0.05)

During the initial 3 days of storage, no discernible difference in TSS content was observed among all guava fruit treatments. However, by day 9, a general decline in TSS content was evident across all treatments, persisting until the conclusion of the 15-day experiment. Notably, non-coated fruits consistently exhibited significantly higher TSS content than those of coated fruits, particularly those treated with 0.75% XG, displaying a reduction in TSS content. On day 15, 0.75% XG-coated fruits demonstrated the lowest TSS (10%), while non-coated fruits exhibited the highest TSS (11%). The TSS content increased throughout the storage period but diminished under the influence of xanthan coatings, following a concentration-dependent pattern (Fig. 2A).

The fruit total titratable acidity (TTA) was markedly influenced by XG coatings, and its trend exhibited an inverse relationship to that of TSS. By day 6, all treatments experienced an increase in TTA content, which continued until the end of experiment. However, the change in TTA content of non-coated fruits was significantly higher than that of coated fruits, where the latter preserved the highest TTA content. On day 15, 0.75% XG-coated fruits displayed the minimum TTA loss (41%), while non-coated fruits exhibited the maximum loss in TTA content (53%) (Fig. 2B). On day 15, 0.75% XG-coated fruits possessed 25% more TTA content than non-coated fruits.

The sugar-acid ratio, representing the ratio between TTS and TTA, exhibited a direct correlation with TSS and increased with the duration of storage. Uncoated fruits on day 15 had the maximum sugar-acid ratio (26.03). Fruits coated with XG showed a 16–26% reduction in the sugar-acid ratio compared to uncoated fruits (Fig. 2C).

The fruit ascorbic acid was significantly affected by the XG coatings and change trend was in line with TTA. During the first 6 days of storage, there was no difference in ascorbic acid content among all studied treatments. By day 9, generally all treatments showed increase in ascorbic acid content that continued till the end of the experiment. However, the change in ascorbic acid content of non-coated fruits was significantly higher than coated fruits, whereas coated fruits retained the highest ascorbic acid content. On day 15, 0.75% XG-coated fruits showed the minimum ascorbic acid loss (22%) whereas the non-coated fruits showed the maximum loss in ascorbic acid content (53%) (Fig. 1D).

### Total phenolics, flavonoids and antioxidants

**Fig. 3:**
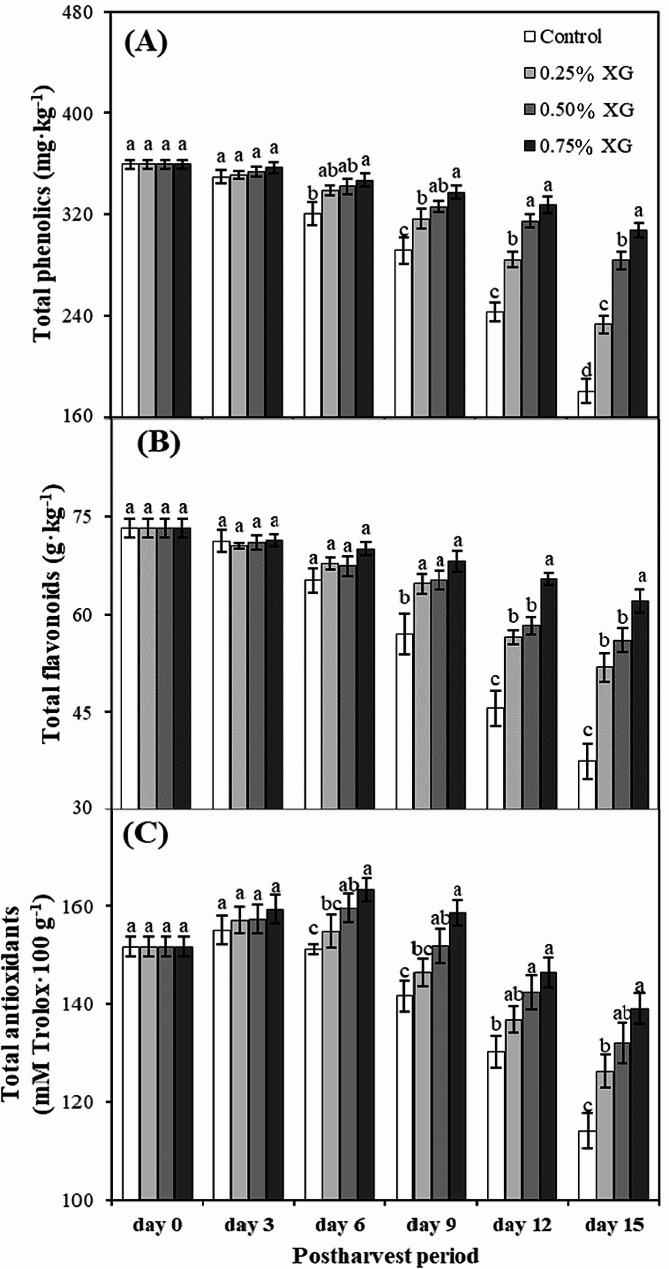
The impact of xanthan gum (XG)-based coatings on total phenolics (**A**), flavonoids (**B**) and antioxidants (**C**) in harvested guava fruits. Each bar corresponds to the mean ± SE (*n* = 4). Statistical analysis using Fisher’s LSD revealed significant differences among coating treatments on each sampling day, denoted by different small letters (*p* ≤ 0.05)

By day 3, there was no significant effect of xanthan treatments on total phenolic content of guava fruits. By day 9, generally all treatments showed decrease in total phenolics till the last day (day 15) of experiment. However, the loss of phenolic content in non-coated fruits was significantly higher than coated fruits, whereas coated fruits, sustained the maximum phenolic, especially 0.75% XG-coated fruits. Compared to total phenolics in guavas on day 0, 0.75% XG-coated fruits showed 15% loss of total phenolics till day 15, which was significantly less than 50% loss of total phenolics shown by non-coated fruits during this duration (Fig. 3A).

The xanthan coating treatment showed non-significant effect on total flavonoids of guava fruits till the 6th day of storage (Fig. 3B). By day 9, generally all treatments showed decrease in flavonoids that continued till the end of the experiment (day 15). However, the change in flavonoid content of non-coated fruits was significantly higher than coated fruits, whereas coated fruits, especially 0.75% XG, preserved the highest flavonoids. On day 15, 0.75% XG-coated fruits showed the minimum flavonoids loss (16%) whereas the non-coated fruit showed the maximum loss in flavonoid contents (49%). On the same day, 0.75% XG-coated fruits had 33% more flavonoids than non-coated ones.

The total antioxidants exhibited a gradual decline in both xanthan gum-treated and control samples throughout the entire storage period (Fig. 3C). Notably, the application of xanthan gum appeared to impede the reduction in antioxidant capacity compared to control fruits, particularly evident after 6 days of storage. Xanthan gum-treated fruits demonstrated a superior antioxidant capacity compared to control fruits by day 9 of storage. By the 15th day, fruits coated with 0.75% Xanthan gum displayed the least loss in antioxidant capacity (6%), while non-coated fruits experienced the maximum reduction (7%).

### Electrolyte leakage, malondialdehyde and hydrogen per oxide content

**Fig. 4:**
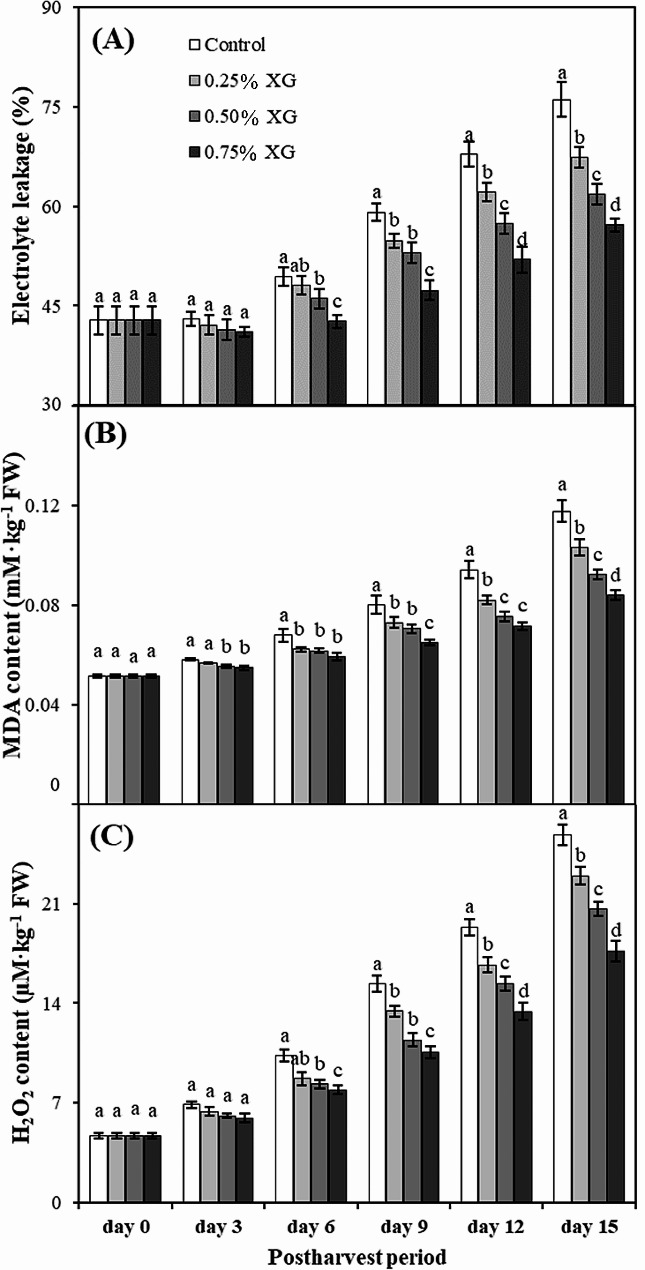
The impact of xanthan gum (XG)-based coatings on electrolyte leakage (**A**), malondialdehyde (**B**) and hydrogen peroxide content (**C**) in harvested guava fruits. Each bar corresponds to the mean ± SE (*n* = 4). Statistical analysis using Fisher’s LSD revealed significant differences among coating treatments on each sampling day, denoted by different small letters (*p* ≤ 0.05)

Membrane leakage, as assessed by electrolyte leakage measurement, exhibited an overall increase during storage across all treatments. However, XG-coated guava fruits demonstrated significantly lower membrane leakage compared to their non-coated counterparts (Fig. 4A). After 15 days of storage, XG-coated fruits showed substantially higher membrane integrity (low electrolyte leakage, i.e., 1.32-fold) than noncoated fruits. On day 15, 0.75% XG-coated fruits showed the minimum increase in electrolyte leakage (13%) whereas the non-coated fruits showed the maximum electrolyte leakage (20%). On the same day, 0.75% XG-coated fruits had 7% less electrolyte leakage than non-coated fruits.

From the 3rd day of storage onward, the content of MDA increased gradually until the conclusion of the storage period (Fig. 4B). However, the change in MDA content of non-coated fruits was significantly higher than that of coated fruits, with XG-coated fruits maintaining the lowest MDA content. On day 15, 0.75% XG-coated fruits exhibited the minimum increase in MDA content (25%), while non-coated fruits showed the maximum increase (46%). On the last day of storage, 0.75% XG-coated fruits had 21% more MDA content compared to non-coated fruits.

The XG treatments showed non-significant effect on H_2_O_2_ content of guava fruits on 3rd day. By day 6, generally all treatments showed increase in H_2_O_2_ content that continued till the end of the experiment. However, the change in H_2_O_2_ content of non-coated fruits was significantly higher than coated fruits, whereas coated fruits, maintained the lowest H_2_O_2_ content. On day 15, 0.75% XG-coated fruits showed the minimum increase in H_2_O_2_ (91%) whereas the non-coated fruits showed the maximum increase in H_2_O_2_ content (138%). On day 15, 0.75% XG-coated fruits had 49% less H_2_O_2_ content than non-coated fruits (Fig. 4C).

### Hemicellulose, cellulose, water soluble pectin and protopectin

**Fig. 5:**
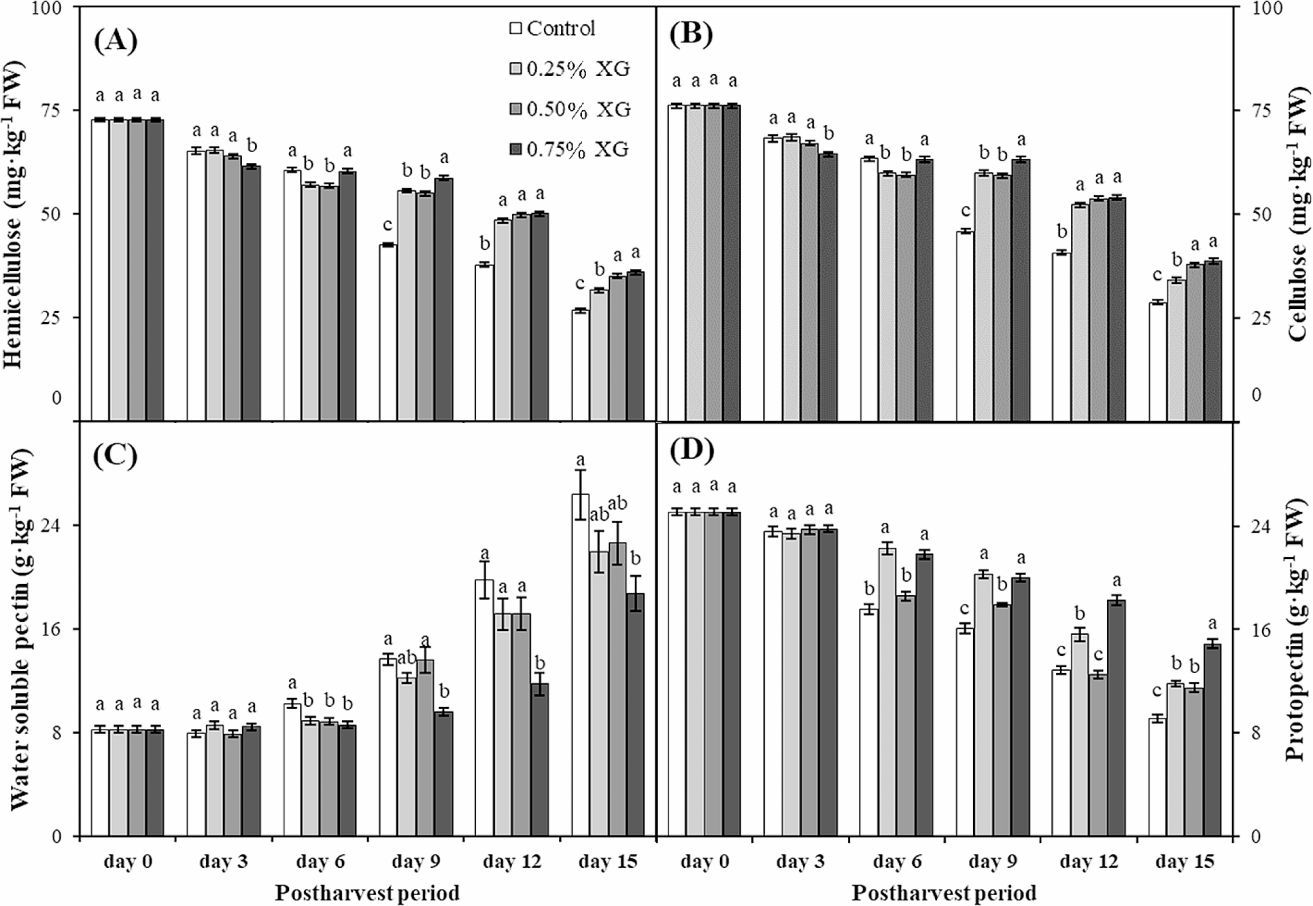
The impact of xanthan gum (XG)-based coatings on hemicellulose (**A**), cellulose (**B**), water soluble pectin (**C**), and protopectin in harvested guava fruits. Each bar corresponds to the mean ± SE (*n* = 4). Statistical analysis using Fisher’s LSD revealed significant differences among coating treatments on each sampling day, denoted by different small letters (*p* ≤ 0.05)

Hemicellulose, cellulose, and protopectin experienced a decline in both coated and uncoated guava fruits as the storage period increased. However, XG coatings effectively maintained higher levels of these components compared to uncoated fruits, thereby preventing the loss of cell integrity in XG-coated fruits. In contrast, the water-soluble pectin (WSP) content increased with the progression of the storage period. A significant (*p* ≤ 0.05) decrease was observed in the hemicellulose and cellulose content of uncoated fruits on the 3rd day of storage, a trend that persisted until the last day of storage (day 15). Guava fruits coated with 0.75% XG exhibited the maximum hemicellulose and cellulose content on the last day of storage (day 15), and this was non-significantly (*p* ≤ 0.05) different from the fruits coated with 0.50% XG (Fig. 5A, B).

Similarly, XG coatings significantly (*p* ≤ 0.05) maintained higher levels of protopectin in guava fruits. On the 15th day of storage, uncoated fruits exhibited the minimum protopectin content (9.10 g·kg^−1^ FW), which was 38.87% lower than the fruits treated with 0.75% XG (14.89 g·kg^−1^ FW) (Fig. 5D). Furthermore, the maximum WSP content (26.38 g·kg^−1^ FW) was found in uncoated fruits on the 15th day of storage. Fruits coated with XG displayed a reduction in WSP content, ranging from 14 to 28% compared to uncoated fruits (Fig. 5C).

### Fruit respiration and ethylene production rates

**Fig. 6:**
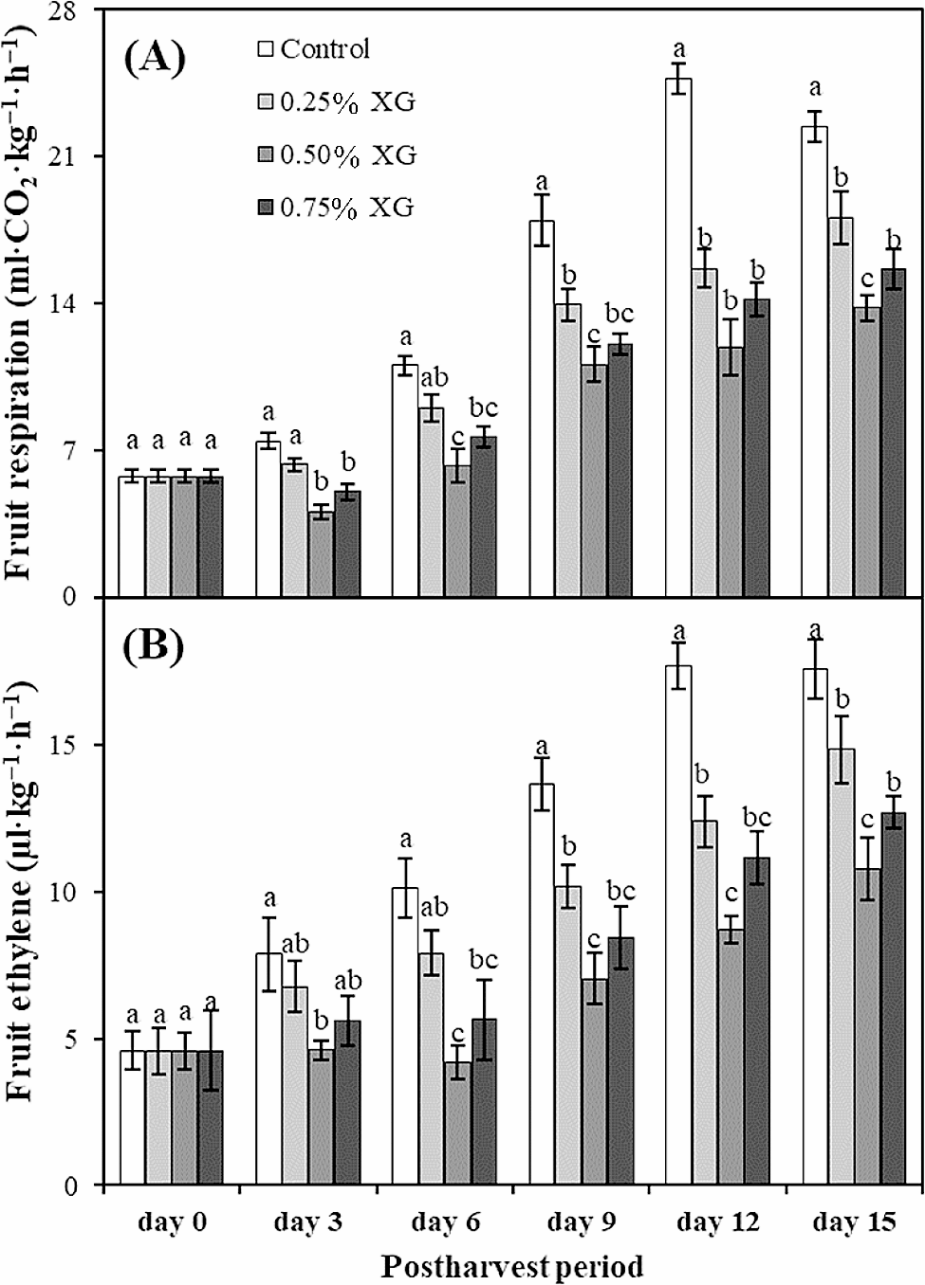
The impact of xanthan gum (XG)-based coatings on fruit respiration (**A**) and ethylene (**B**) rate in harvested guava fruits. Each bar corresponds to the mean ± SE (*n* = 4). Statistical analysis using Fisher’s LSD revealed significant differences among coating treatments on each sampling day, denoted by different small letters (*p* ≤ 0.05)

Regardless of coating applied, CO_2_ and ethylene production rates exhibited an increase over the storage period. However, in comparison with the control, XG coatings consistently maintained reduced levels of respiration and ethylene throughout the entire storage period. Notably, a significant (*p* ≤ 0.05) change in fruit respiration was observed 3 days after coating, particularly in fruits treated with 0.50–0.75% XG. Moreover, on the 15th day, XG-coated guava fruits displayed a remarkably reduced respiration rate compared to uncoated fruits. On the same day (day 15), the minimum respiration rate was recorded in fruits coated with 0.50% XG, representing a 38.43% reduction compared to uncoated fruits (Fig. 6A).

Similarly, in the case of fruit ethylene, XG coatings maintained significantly (*p* ≤ 0.05) less ethylene levels in guava fruits compared with uncoated fruits. On the 15th day, uncoated fruits exhibited the maximum ethylene rate (17.59 µl·kg−^1^·h^−1^), which was 38.48% higher than the fruits treated with 0.75% XG (12.70 µl·kg^−1^·h^−1^) (Fig. 6B).

### Activities of ROS-scavenging enzymes

**Fig. 7:**
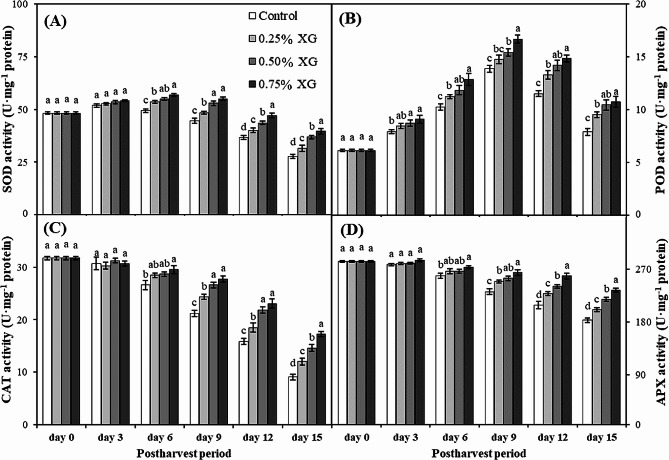
The impact of xanthan gum (XG)-based coatings on activities of ROS-scavenging enzymes i.e., SOD (**A**), POD (**B**), CAT (**C**), and APX (**D**) in harvested guava fruits. Each bar corresponds to the mean ± SE (*n* = 4). Statistical analysis using Fisher’s LSD revealed significant differences among coating treatments on each sampling day, denoted by different small letters (*p* ≤ 0.05)

Fruits coated with xanthan gum exhibited higher enzymatic activities than control fruits, indicating that xanthan gum coating effectively delayed the senescence of guava fruits. During the initial 3 days of storage, there was no significant difference in SOD activity among all coating treatments. However, by the 6th day of storage, xanthan coating significantly improved SOD activity in the fruit pulp of guava. As storage progressed, a general decrease in SOD activity was observed across all treatments until the end of the experiment (day 15). Notably, the inhibition of SOD activity in non-coated fruits was significantly higher than in coated fruits, with 0.75% XG coating exhibiting the maximum SOD activity. The SOD activity demonstrated a concentration-dependent decrease during storage under the influence of XG coating (Fig. 7A).

Regarding POD activity, all treatments generally showed an increase until the 9th day, followed by a decrease on the 12th and 15th days. On the 9th day, 0.75% XG-coated fruits displayed the maximum POD activity, representing a 62% increase compared to non-coated fruits (Fig. 7B). The POD activity in guava fruits markedly increased within the first 9 days of storage and then decreased during the latter part of storage.

During the initial 3 days of storage, there was no significant difference in CAT activity among all coating treatments. However, by the 9th day, a general decrease in CAT activity was observed across all treatments, continuing until the end of the experiment (day 15). The reduction in CAT activity in non-coated fruits was significantly higher than in coated fruits, especially 0.75% XG, which preserved the highest CAT activity. On day 15, 0.75% XG-coated fruits showed the minimum CAT reduction (45%), while non-coated fruits exhibited the maximum decrease in CAT content (70%). The CAT activity decreased progressively during storage and was retained under the influence of xanthan coating in a concentration-dependent manner. The lowest CAT activity was recorded in non-coated fruits on the 15th day of cold storage (Fig. 7C).

On the 6th day of storage, xanthan treatments significantly influenced APX activity in guava fruits. By the 9th day, all treatments exhibited a decrease in APX content, continuing until the end of the experiment (day 15). However, the loss in APX content in non-coated fruits was significantly higher than in coated fruits. On the 15th day of storage, 0.75% XG-coated fruits showed the minimum reduction in APX activity (6%), while non-coated fruits exhibited the maximum loss of APX activity (15%) (Fig. 7D).

### Activities of fruit softening enzymes

**Fig. 8:**
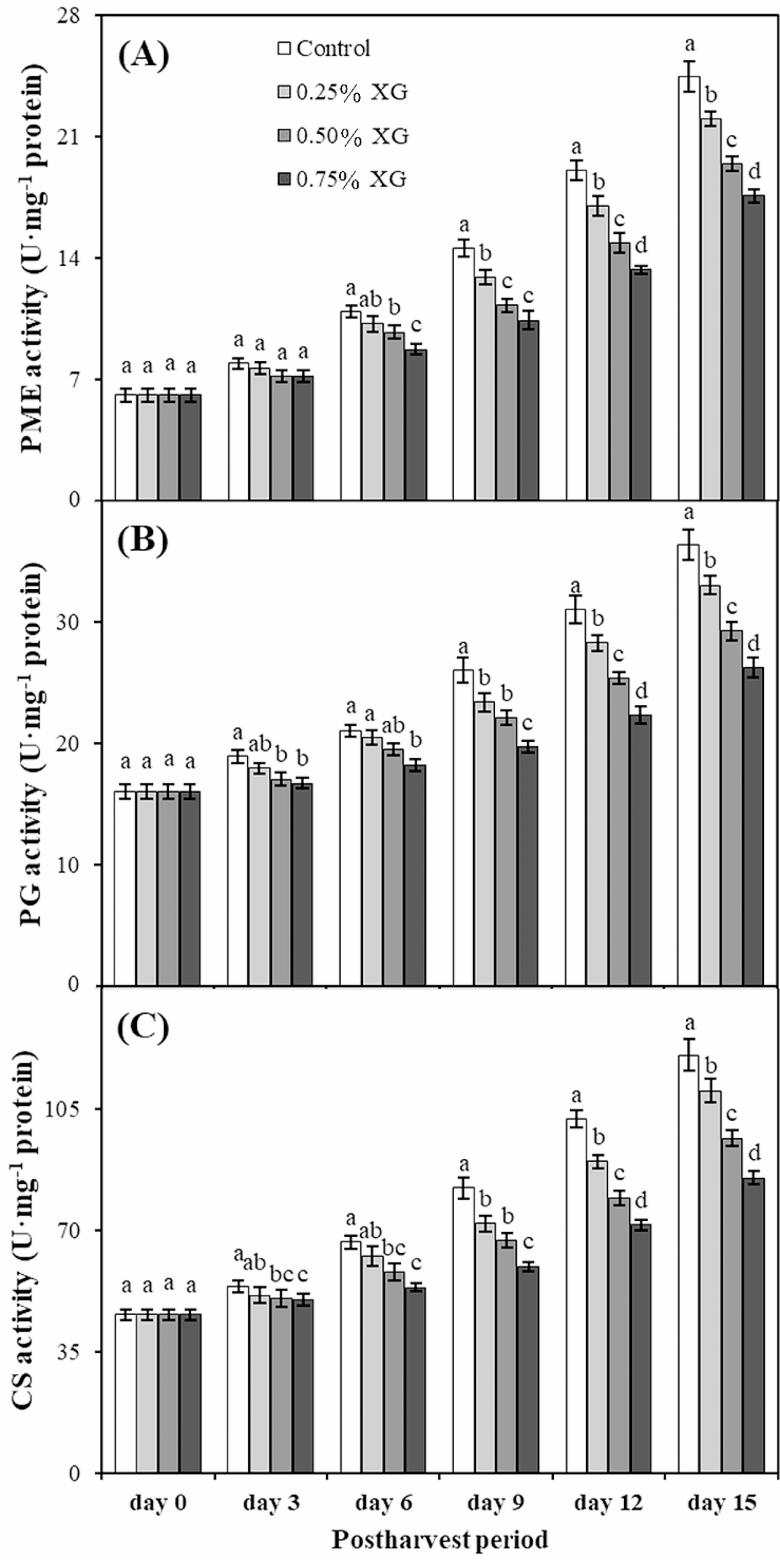
The impact of xanthan gum (XG)-based coatings on activities of fruit softening enzymes i.e., pectin methylesterase (**A**), polygalacturonase (**B**), and cellulase (**C**) in harvested guava fruits. Each bar corresponds to the mean ± SE (*n* = 4). Statistical analysis using Fisher’s LSD revealed significant differences among coating treatments on each sampling day, denoted by different small letters (*p* ≤ 0.05)

In general, the activities of fruit softening enzymes, namely PME, PG, and CS, increased in both coated and uncoated guava fruits with the extended storage duration. However, XG coatings consistently mitigated their levels throughout the entire storage period in comparison with the control. Specifically, the highest PME activity (24.48 U·mg^−1^ protein) was observed in uncoated fruits on the 15th day of storage. Guava fruits coated with XG exhibited a reduction in PME activity ranging from 9 to 28% compared to uncoated fruits (Fig. 8A).

Similarly, on the 15th day of storage, uncoated fruits displayed the maximum PG activity (36.38 U·mg^−1^ protein), which was 38.51% higher than the PG activity in fruits treated with 0.75% XG (26.27 U·mg^−1^ protein) (Fig. 8B). Furthermore, a significant (*p* ≤ 0.05) reduction was observed in cellulase activity in fruits treated with 0.50% and 0.75% XG on the 3rd day of storage, and this trend persisted until the end of the experiment. On the 15th day, irrespective of the applied concentration, XG coating exhibited remarkably reduced CS activity compared to no coating in stored guava fruits. The minimum CS activity (85.20 U·mg^−1^ protein) was recorded in fruits coated with 0.75% XG, representing a 29.31% reduction compared to uncoated fruits (120.54 U·mg^−1^ protein) (Fig. 8C).

### Organoleptic and sensory evaluation

Overall, the scores for aroma, taste, and overall acceptability of both XG-coated and uncoated guava fruits demonstrated improvement with the extended storage time period. Notably, the highest scores for aroma, taste, and acceptability were attained on the 15th day of storage in guava fruits coated with 0.75% XG, followed by the fruits coated with 0.50% XG (Table 1).

**Table 1:** The impact of xanthan gum (XG)-based coatings on aroma, taste and overall acceptability of harvested guava fruits stored for 15 days at 20 ± 1 °C and 85–90% RH.

	Treatments	Day 0	Day 3	Day 6	Day 9	Day 12	Day 15
**Aroma**	Control	6.16 ^a^	6.58 ^a^	6.83 ^a^	7.75 ^a^	7.75 ^a^	6.75 ^c^
	0.25% XG	6.16 ^a^	6.25 ^a^	6.58 ^a^	7.25 ^b^	7.66 ^a^	7.33 ^b^
	0.50% XG	6.16 ^a^	6.25 ^a^	6.25 ^a^	6.83 ^b^	7.5 ^a^	7.75 ^ab^
	0.75% XG	6.16 ^a^	6.25 ^a^	6.41 ^a^	6.91 ^b^	7.41 ^a^	7.91 ^a^
**Taste**	Control	5.25 ^a^	6.16 ^a^	6.75 ^a^	7.41 ^a^	7.66 ^a^	6.83 ^c^
	0.25% XG	5.25 ^a^	5.91 ^a^	6.33 ^b^	7.25 ^ab^	7.75 ^a^	7.41 ^b^
	0.50% XG	5.25 ^a^	5.83 ^a^	6.08 ^b^	6.66 ^b^	7.5 ^a^	7.66 ^ab^
	0.75% XG	5.25 ^a^	5.75 ^a^	6.16 ^b^	6.75 ^b^	7.41 ^a^	7.91 ^a^
**Acceptability**	Control	6.25 ^a^	6.58 ^a^	7 ^a^	7.75 ^a^	7.75 ^a^	6.66 ^c^
	0.25% XG	6.25 ^a^	6.25 ^a^	6.58 ^ab^	7.25 ^b^	7.75 ^a^	7.41 ^b^
	0.50% XG	6.25 ^a^	6.25 ^a^	6.25 ^b^	6.75 ^c^	7.5 ^a^	7.66 ^ab^
	0.75% XG	6.25 ^a^	6.25 ^a^	6.41 ^b^	6.91 ^bc^	7.41 ^a^	7.91 ^a^

Statistical analysis using Fisher’s LSD revealed significant differences among coating treatments on each sampling day, denoted by different small letters (*p* ≤ 0.05).

### Pearson (*n*) correlation

**Fig. 9:**
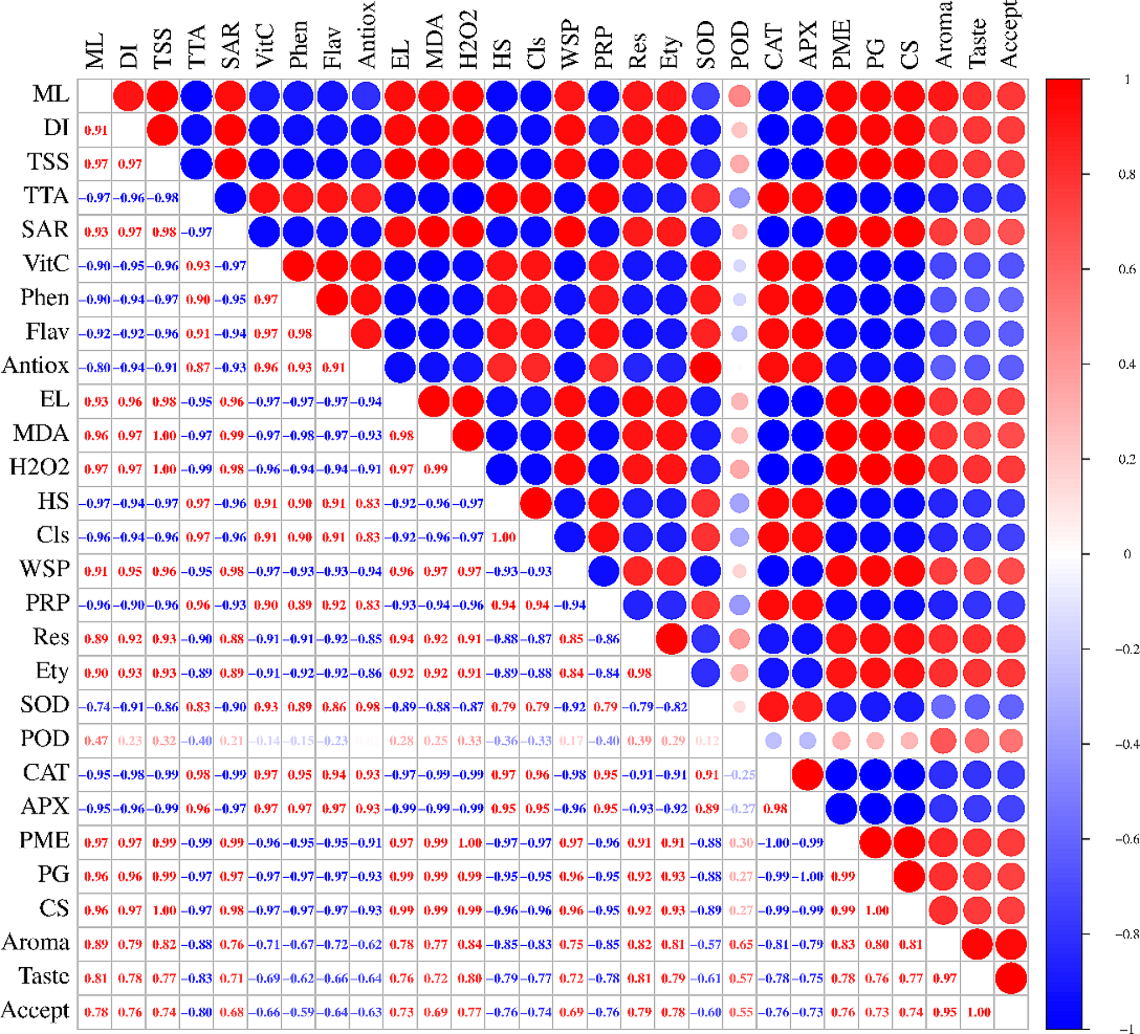
Pearson (*n*) correlation among different studied parameters of guava fruits. Abbreviations: ML – moisture loss; DI – decay incidence; TSS – total soluble solids; TTA – total titratable acidity; SAR – sugar-acid ratio; VitC – ascorbic acid; Phen – total phenolics; Flav – total flavonoids; Antiox – total antioxidants; EL – electrolyte leakage; MDA – malondialdehyde content; H2O2 – hydrogen peroxide; HS – hemicellulose; Cls – cellulose; WSP – water soluble pectin; PRP – protopectin; Res – respiration; Ety – ethylene; SOD – SOD activity; POD – POD activity; CAT – CAT activity; APX – APX activity; PME – PME activity; PG – PG activity; CS – CS activity; Accept – overall acceptability

Fruit moisture loss demonstrated a robust positive correlation with decay incidence (Fig. 9). Additionally, both moisture loss and decay incidence exhibited positive associations with indicators of oxidative stress, including electrolyte leakage, MDA content, and H_2_O_2_ content, as well as with factors such as respiration, ethylene, and enzymes contributing to fruit softening. Conversely, there was a negative correlation with enzymes such as SOD, CAT, and APX, as well as with total phenolics, flavonoids, antioxidants, hemicellulose, cellulose, and protopectin. The relationship between the fruit TSS and TTA revealed a negative correlation. Total phenolics, flavonoids, and antioxidants demonstrated positive correlations with TTA, ascorbic acid, cellulose, hemicellulose, water-soluble pectin, and ROS-scavenging enzymes but showed a negative relationship with fruit softening enzymes. Electrolyte leakage, MDA, and H_2_O_2_ exhibited positive relationships with respiration, ethylene, and fruit softening enzymes but negative correlations with antioxidant activities. SOD, CAT, and APX were positively associated with antioxidants, cellulose, and hemicellulose and negatively correlated with oxidative stress indicators and fruit softening enzymes. On the other hand, the organoleptic attributes of the fruit were positively correlated with moisture loss, TSS, the sugar-acid ratio, oxidative stress indicators, WSP, respiration, ethylene, and fruit softening enzymes (Fig. 9).

## Discussion

Postharvest moisture loss poses a significant challenge, resulting in notable losses of fresh fruits and vegetables [42]. This issue arises as the harvested produce tends to dry out, necessitating effective management to minimize the decline in quality. The consequences of unchecked moisture loss include fruit shriveling, as observed in guavas, ultimately diminishing their market value [43]. In the course of this study, it was noted that guavas coated with xanthan gum exhibited a noteworthy reduction in moisture loss compared to their uncoated counterparts. Edible coatings, such as xanthan gum, are widely acknowledged for their ability to create a protective layer that mitigates moisture loss, thereby preventing dehydration and transpiration. Consequently, there is a substantial decrease in the amount of moisture lost when compared to that in plants lacking such protective coatings. The findings of this study underscore the effectiveness of xanthan gum in forming a protective layer on guavas, which leads to diminished moisture loss in comparison to that of uncoated fruit. Additionally, other research has highlighted similar benefits, demonstrating that grapes [44] coated with xanthan gum and jujube also experience reduced moisture loss [20].

Postharvest pathological infections significantly contribute to the spoilage of fresh fruits and vegetables during storage, emphasizing the need for environmentally friendly management methods. Edible coatings have gained widespread recognition as eco-friendly options for addressing postharvest diseases in fruits [45]. In the context of this study, guavas treated with a protective coating exhibited a reduction in decay incidence. This decrease in decay incidence in the coated guava fruit can be attributed to the xanthan gum-based coating, which served as a protective barrier against disease-causing pathogens, resulting in a significant decrease in decay. Supporting evidence from a study by Prasad et al. [46] revealed that mango fruit coated with xanthan gum experienced a decrease in both decay and disease incidence. Compared with those treated with carboxymethyl cellulose and guar gum, the mango plants treated with xanthan gum exhibited fewer instances of anthracnose and stem end rot. Furthermore, in another investigation, Wani et al. [17] observed a reduction in decay incidence among strawberry fruits coated with xanthan gum compared to both the uncoated control and treatments using a gum arabic coating.

The quality attributes of guava fruits, including the TSS, TTA, and sugar-acid ratio, play crucial roles in assessing maturity and ripening. Over the storage period, the concentrations of TSS and TTA in guava fruit flesh change, with TSS tending to increase while TTA decreases [43]. These alterations contribute to an elevated sugar‒acid ratio, providing insights into fruit maturity and ripening [47]. In the course of this study, a consistent increase in the TSS and the sugar-acid ratio was noted, accompanied by a gradual decrease in the TTA. It is plausible that the application of a xanthan gum coating played a role in averting moisture loss and slowing the aging process, resulting in a slower and smaller increase in TSS content. The enhanced preservation of TTA content in guavas coated with xanthan gum may be attributed to reduced metabolic activity, preventing the depletion of organic acids and maintaining higher TTA levels during storage. A study by Kumar et al. [48] demonstrated a similar response in mango fruit coated with xanthan gum, in which a reduced increase in TSS, a delayed reduction in TTA, and a suppressed sugar‒acid ratio were observed. Ascorbic acid, a vital antioxidant compound with nonenzymatic properties, has been highlighted in recent studies [49–51]. The uncoated fruit exhibited a rapid reduction in ascorbic acid content throughout storage, a phenomenon mitigated by the application of xanthan gum. Notably, ascorbic acid is susceptible to autoxidative degradation during postharvest storage [48, 49]. Edible coatings on fruit can alter the internal atmospheric composition, leading to a reduction in oxygen availability [52]. From an agronomist’s perspective, it is likely that the xanthan gum coating significantly influenced the oxygen uptake of guavas. This, in turn, resulted in a noteworthy decrease in the oxidative degradation of ascorbic acid, ultimately maintaining a higher concentration of this compound than that in uncoated fruit.

Upon harvest, fruits are susceptible to oxidative stress; however, their innate defense mechanism involves the use of nonenzymatic antioxidants, such as phenols and flavonoids, to counteract this stress [53, 54]. This study revealed that guavas coated with xanthan gum exhibited preserved levels of total phenolics, flavonoids, and antioxidants in comparison to those in the control group. Edible coatings have proven effective at inhibiting oxidation, leading to heightened levels of flavonoids and phenols. Moreover, it is probable that the coated fruit possessed a more robust structure, as evidenced by the reduced electrolyte leakage. This structural fortification likely restricted the interaction between polyphenol oxidase (PPO enzyme, responsible for fruit discoloration) and flavonoids and phenols, distinguishing this interaction from the dynamics observed in uncoated fruits. Studies have indicated that edible coatings can safeguard the structural integrity of fruits, preventing direct contact between specific compounds [55]. This preservation contributes to maintaining elevated levels of nonenzymatic antioxidants, such as phenols and flavonoids, as evidenced in a study on carboxymethylcellulose-coated fresh-cut ‘Maharaji’ apples [56].

Comprehending the respiration rate and ethylene production is paramount for determining the maturation, ripening, and softening processes of fruits. Throughout this study, these metabolic activities exhibited an initial increase until the 12th day of storage, followed by a subsequent decrease. This observed trend aligns with the findings of Chen et al. [52], who similarly noted a comparable pattern in ethylene biosynthesis and respiration rate in jujubes. The application of edible coatings is critical for creating a thin layer on the fruit surface, inducing changes in the produce by elevating CO_2_ levels and diminishing the accumulation of internal O_2_. Consequently, there is a notable reduction in ethylene production and the respiration rate [57, 58]. It appears that xanthan gum may have influenced the internal atmosphere of the fruit, leading to heightened CO_2_ levels and reduced O_2_ levels. This alteration resulted in decreased rates of respiration and ethylene production. Consistent with our research, a significant decrease in ethylene production and the respiration rate was also observed in mango fruit coated with xanthan gum [46, 48].

Understanding the interplay between cellular membrane integrity, lipid peroxidation metabolism, and the rate of ROS production during guava storage is of paramount importance [59]. Elevated levels of ROS can adversely affect cell membrane stability, triggering lipid peroxidation and generating the cytotoxic metabolite MDA [60]. Suppression of increased ROS production becomes crucial for curtailing lipid peroxidation, preventing the disruption of dynamic equilibrium and subsequent increases in membrane permeability. This, in turn, leads to heightened electrolyte leakage, a pivotal factor in fruit senescence and softening [61]. In the present study, guavas coated with xanthan gum exhibited lower levels of electrolyte leakage and malondialdehyde (MDA) than did uncoated fruits. The decreases in electrolyte leakage and MDA levels can likely be ascribed to the reduction in ROS generation, which results in the suppression of fruit senescence and ripening. Preserving membrane integrity ultimately led to a decrease in the MDA concentration. H_2_O_2_, generated through oxidative metabolism, is associated with rapid fruit senescence during postharvest [62]. In this study, the concentration of H_2_O_2_ was significantly lower in the guavas coated with xanthan gum compared to the uncoated group. It is believed that the application of xanthan gum contributes to maintaining the activity of the antioxidative system, resulting in delayed guava fruit senescence and a reduction in softening.

The accumulation of ROS typically leads to an imbalance between scavenging and production, causing damage to membrane integrity and accelerating the senescence and softening of guava fruits [59]. Therefore, it is crucial to promptly scavenge overproduced ROS molecules to delay the rapid ripening and softening of guava fruits. Antioxidant enzymes, including SOD, POD, CAT, and APX, collaborate to eliminate harmful ROS molecules, effectively preventing excessive softening of fruit tissues [6]. SOD plays a pivotal role in combating oxidative stress by converting O_2_^–•^ into O_2_ and H_2_O. Conversely, CAT and APX breakdown H_2_O_2_ into O_2_ and H_2_O. Similarly, POD functions as an oxidoreductase, contributing to the breakdown of H_2_O_2_ into O_2_ and H_2_O [63]. In the present study, compared with those in the control group, the application of the xanthan gum coating stimulated the activities of SOD and POD while also maintaining higher levels of CAT and APX activities.

The hemicellulose-cellulose network plays a pivotal role in preserving the adhesion and turgidity of the cell-to-cell configuration [64]. During storage, the concentration of WSP increased, while the levels of hemicellulose, cellulose, and protopectin gradually decreased, as evidenced in this study. These changes in cell wall components can lead to a reduction in cell wall strength, resulting in cell separation and the loss of intracellular adhesion. This progression contributes to the timely softening of fruits [65]. It is apparent that xanthan gum effectively curbed the increase in WSP, concurrently promoting the enhanced preservation of hemicellulose, cellulose, and protopectin. The delayed solubilization and subsequent depolymerization of polysaccharide contents in cell walls likely contributed to maintaining higher firmness and postponing the onset of guava fruit softening.

Several cell wall hydrolases, including PME, PG, and CS, collaboratively regulate the breakdown of cell wall components associated with softening. PME plays a crucial role in pectin methylation, while the PG compromises the integrity of the middle lamella of fruit tissues, leading to excessive fruit softness [66]. In the present study, xanthan gum-based treatment inhibited the increase in PME and PG activity. The reduced activities of PME and PG lessened the rapid increase in WSP, suggesting that coating application effectively delayed the breakdown of pectin. Similarly, xanthan gum-coated strawberries [17] and mangoes [46] exhibited significantly reduced PME and PG enzyme activity. The breakdown of the xyloglucan network into hemicellulose and cellulose, affecting the structural integration of the cell wall, is mainly attributed to the CS enzyme. The CS enzyme can degrade both cellulose and xyloglucan, the primary components of the cellulose-hemicellulose network [67]. As the levels of cellulose and hemicellulose decreased, our findings indicated an increase in CS activity in guava fruit over time. However, the xanthan gum coating inhibited the action of CS, likely contributing to the preservation of cellulose and hemicellulose and delaying the softening of guava fruit. In line with our results, compared with those in the control group, the fruits in the xanthan gum-coated strawberry treatment group exhibited decreased CS enzyme activity, preserving greater firmness [17].

## Conclusion

This study underscores the remarkable efficacy of xanthan gum coatings in safeguarding the postharvest quality of guava fruit. The protective barrier formed by xanthan gum addresses key challenges such as moisture loss and decay incidence, contributing to prolonged shelf life and sustained market value. The coating’s positive impact on physiological attributes, including the TSS, TTA, sugar-acid ratio, and ascorbic acid content, signifies its role in preserving nutritional content. Additionally, the research highlights the influence of the coating on metabolic activities, antioxidant enzymes, and the hemicellulose-cellulose network, collectively contributing to the reduction in ROS production, electrolyte leakage, and membrane disruption. These findings not only deepen our understanding of the underlying mechanisms involved but also present xanthan gum as an environmentally friendly and promising solution for enhancing the postharvest resilience of guava fruits, with implications for sustainable practices in the fruit industry.

## Data Availability

No datasets were generated or analysed during the current study.
